# Diplopia and tongue deviation following halo-pelvic traction for severe congenital scoliosis: a case report

**DOI:** 10.3389/fped.2026.1698138

**Published:** 2026-03-31

**Authors:** Rui Liu, Hui Zhou, Na Li, Yulian Chen

**Affiliations:** 1 Department of Rehabilitation Medicine, West China Second University Hospital, Sichuan University, Chengdu, Sichuan, China; 2 Department of Rehabilitation Medicine, WCSUH-Tianfu Sichuan Provincial Children's Hospital, Sichuan University, Meishan, Sichuan, China

**Keywords:** congenital scoliosis, halo-pelvic traction, cranial nerve injury, case report, complication

## Abstract

**Objective:**

To report a case of diplopia and tongue deviation following halo-pelvic traction (HPT) in a patient with severe congenital scoliosis (CS) and to analyze the potential causes.

**Methods:**

A retrospective analysis of the patient's clinical data was conducted, including medical history, physical examination, imaging findings, treatment course, and outcomes.

**Results:**

A 12-year-old female with CS was admitted. After 40 days of traction (with a total longitudinal lengthening of approximately 12 cm), she developed painless horizontal diplopia and leftward deviation of the protruded tongue. Physical examination revealed limited abduction of the left eye and leftward deviation of the protruded tongue. Traction was discontinued, and neurotrophic therapy (including corticosteroids, mecobalamin, and B-complex vitamins) was initiated. Symptoms gradually resolved within 50 days, with complete resolution of diplopia and normalized tongue protrusion.

**Conclusion:**

Early recognition and discontinuation of traction, combined with multimodal therapy, may prevent permanent cranial nerve injury in patients undergoing HPT.

## 1 Introduction

Congenital scoliosis (CS) is a complex three-dimensional spinal deformity that originates from embryological malformations of the vertebral column (1). Its prevalence is estimated at 0.5 to 1 per 1,000 live births, accounting for approximately 10% of scoliosis cases (2). Notably, 30%–60% of affected individuals exhibit concomitant anomalies of the cardiovascular, genitourinary, or gastrointestinal systems (3). Halo-pelvic traction (HPT) is an established technique for managing severe CS. Previous studies demonstrate its efficacy in improving spinal deformity and pulmonary function, significantly enhancing the correction rate and safety of subsequent surgeries (4–6). However, HPT carries risks such as pin-site infection and cervical stiffness (4). Although direct cranial nerve injury (CNI) is a recognized but rare complication of HPT (7–9), previous reports have primarily described palsies of the abducens (VI) and hypoglossal (XII) nerves. The pathophysiological mechanisms remain incompletely defined. This report adds new knowledge by describing simultaneous abducens and hypoglossal nerve involvement during HPT, a rare combination that highlights the need for vigilant neuromonitoring. By integrating our observations with existing literature, we elucidate potential mechanisms and underscore the clinical importance of this complication.

## 2 Case report

**Figure 1:**
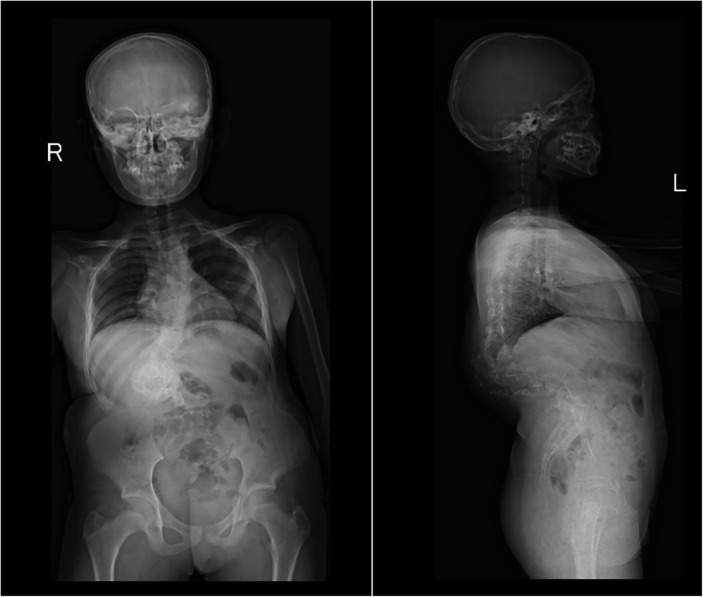
Preoperative full-length spine radiographs (anteroposterior and lateral views) showing S-shaped scoliosis.

A 12-year-old girl was admitted for progressive spinal deformity noted since early childhood. There was no antecedent trauma, prior surgery, or family history of musculoskeletal or connective-tissue disorders. Height was 133 cm and weight 40.3 kg. Physical examination revealed a prominent thoracolumbar “razor-back” deformity, shoulder imbalance, and equal leg lengths. Standing full-length anteroposterior and lateral radiographs of the spine demonstrated an S-shaped double major curve: a left upper-thoracic curve with the apex at T6 measuring 40° Cobb, and a right thoracolumbar curve measuring 62° Cobb (Figure 1). Pulmonary function tests indicated small-airway obstruction and increased airway resistance. CT angiography of the chest and abdomen, echocardiography, and electrocardiography were normal.

**Figure 2:**
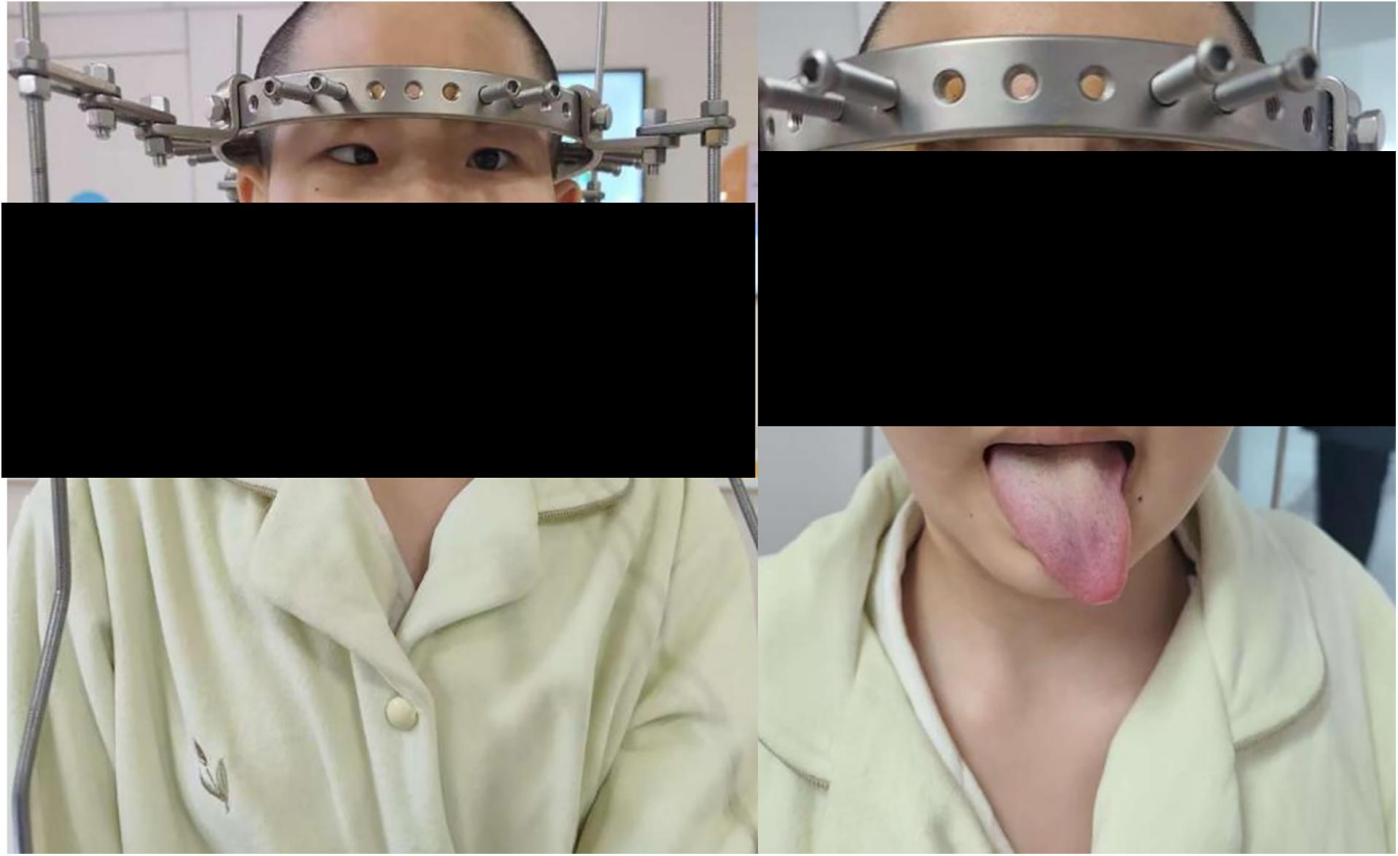
After 40 days of HPT, demonstrating limited left eye abduction (arrow) and leftward tongue deviation (arrow).

After HPT was initiated, the patient was transferred to pediatric rehabilitation for pulmonary conditioning and motor training, including respiratory muscle strengthening and lung volume expansion exercises. The traction protocol involved gradual distraction based on patient tolerance and radiographic monitoring. Specifically, distraction was advanced at a rate of 15–20 mm per week during the first month, and at 6–10 mm per week during the second month. On post-admission day 40, after achieving approximately 12 cm of total vertical elongation, she developed painless horizontal diplopia and leftward tongue deviation (Figure 2).

Vital signs remained stable throughout, with no evidence of elevated intracranial pressure. Neurological examination revealed limited abduction of the left eye (cranial nerve VI injury) and leftward tongue deviation (cranial nerve XII injury). Cranial nerve assessment is summarized in Table 1. Electroneurography of the facial nerve demonstrated reduced compound muscle action potential (CMAP) amplitudes with prolonged distal latencies in the marginal mandibular and zygomatic branches, confirming axonal involvement. Brain CT excluded intracranial hemorrhage, infarction, or mass lesions. MRI was not performed due to the halo device, limiting detailed cranial nerve visualization.

**Table 1:** Cranial nerve examination findings.

Cranial nerve	Function tested	Findings	Interpretation
II	Visual acuity	Normal	No injury
III, IV, VI	Extraocular movements	Limited left eye abduction	CN VI palsy
V	Facial sensation	Normal	No injury
VII	Facial symmetry	Normal	No obvious injury
IX, X	Gag reflex, swallowing	Normal	No injury
XII	Tongue protrusion	Leftward deviation	CN XII palsy

Given the ocular and lingual deficits, we hypothesized a traction-induced cranial neuropathy affecting the left abducens (VI) and hypoglossal (XII) nerves, most likely of mechanical origin. The halo-pelvic construct was immediately shortened by 3 cm, and traction was suspended. Low-dose oral corticosteroids, mecobalamin, and B-complex vitamins were started, complemented by acupuncture. Two weeks later, left-eye excursion had improved and repeat neurophysiology showed increased CMAP amplitudes; confirming clinical recovery. Consequently, corticosteroids were discontinued, while mecobalamin and B-vitamin supplementation were maintained.

**Figure 3:**
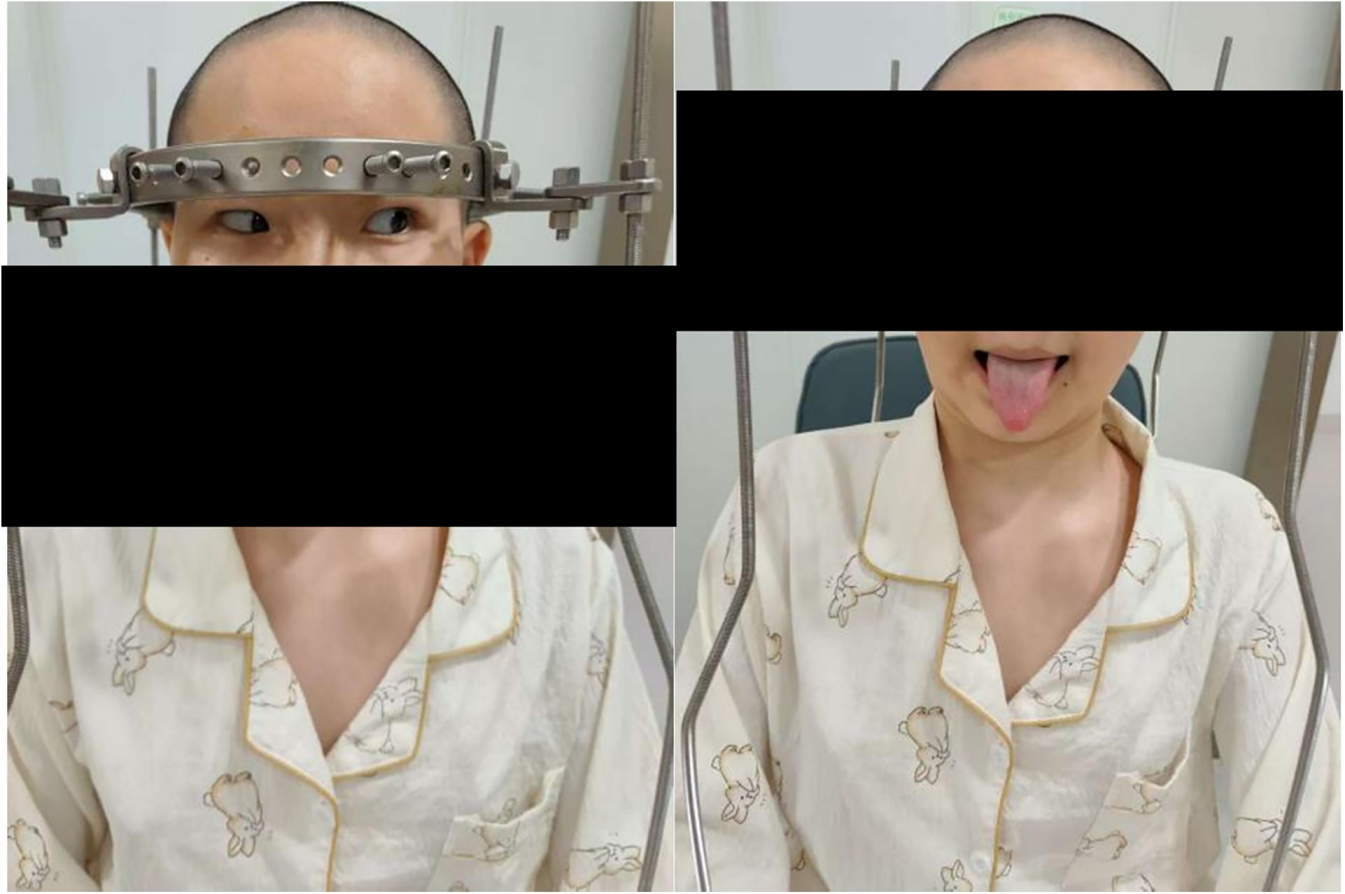
At 50-day follow-up, showing improved eye abduction and midline tongue protrusion (arrows).

At the 50-day assessment, the patient reported complete resolution of diplopia. Physical examination revealed a standing height of 145 cm, full abduction of the left eye, and midline tongue protrusion (Figure 3). She was subsequently transferred to pediatric orthopaedics for a second-stage posterior spinal release. A Ponte osteotomy with segmental pedicle-screw instrumentation was performed uneventfully. Post-operatively, the patient continued to report clear vision; ocular motility was normal in all fields of gaze, and tongue position remained central.

Following this procedure, the patient completed a two-month course of intensive pulmonary rehabilitation and motor function training. Subsequently, she received the definitive posterior spinal correction with segmental instrumentation and was discharged uneventfully. Regular telephone follow-ups have been conducted since discharge. At the most recent follow-up, five months after the initial onset of symptoms, there has been no recurrence of diplopia or tongue deviation.

## 3 Discussion

HPT is an effective treatment for CS, with reported complication rates ranging from 6.6% to 26.7%. Major complications include pin tract infection, atlantoaxial instability, and neurological symptoms in the lower limbs, among others (4, 10). The diplopia and tongue deviation observed in the present case constitute exceptionally rare cranial neuropathies whose precise pathogenesis remains incompletely defined. While previous publications have documented similar complications, predominantly involving the abducens nerve (VI), and glossopharyngeal (IX), vagus (X), or hypoglossal (XII) nerves in association with halo-traction, the involvement of the facial nerve (VII) as suggested by electrophysiological findings in our case is particularly unusual and has not been extensively reported (7–9). It is noteworthy that patients with myelomeningocele are considered at higher risk for such traction-related neurological complications. In the present case, myelomeningocele was definitively ruled out based on preoperative physical examination and imaging studies.

Current hypotheses center on several potential mechanisms: (1) direct mechanical stretch transmitted to cranial nerves by sustained axial traction; (2) entrapment or compression of nerves at anatomically fixed osteofibrous tunnels or dural foramina; (3) vascular compromise due to traction-induced ischemia or reduced blood flow to the nerves; and (4) alterations in cerebrospinal fluid dynamics, which may affect nerve function through changes in pressure or perfusion. While direct mechanical stretch is the most commonly cited mechanism, vascular and fluid dynamic factors should also be considered, particularly in prolonged traction. The abducens nerve (VI) is particularly vulnerable because of its long intracranial course and relative fixation at Dorello's canal, where the nerve is anchored by the petroclinoid ligament within a narrow osseous conduit (11). Axial traction can therefore generate focal shear or compressive forces at this site. The asymmetric tongue protrusion is a classic sign of hypoglossal nerve (XII) injury. However, the concomitant electroneurography (ENoG) findings demonstrating reduced compound muscle action potential amplitudes and prolonged distal latencies in the marginal mandibular and zygomatic branches of the facial nerve (VII) provide objective evidence of concurrent axonal involvement of these specific branches. This presents a diagnostic challenge. While the hypoglossal nerve (XII) is primarily responsible for tongue movement and protrusion, injury to the marginal mandibular branch of the facial nerve (VII)—which innervates muscles such as the depressor anguli oris and mentalis—could disrupt the muscular equilibrium of the floor of the mouth and lower lip. This imbalance, particularly weakness of the ipsilateral mentalis muscle that stabilizes the tongue base during protrusion, might accentuate the appearance of tongue deviation when combined with a hypoglossal nerve injury. Therefore, although the primary cause is likely hypoglossal nerve damage, the facial nerve involvement may contribute to the clinical presentation. Nevertheless, a primary hypoglossal nerve lesion remains the most plausible cause for the dominant tongue deviation.

Similarly, the marginal mandibular and zygomatic branches of the facial nerve (**VII**) are susceptible to traction-induced injury. After traversing the fallopian canal within the temporal bone, the facial nerve exits the skull base through the stylomastoid foramen. The marginal mandibular branch supplies the depressor anguli oris and platysma, whereas the zygomatic branch innervates the orbicularis oculi and levator labii superioris. Prolonged axial loading may stretch or compress the facial nerve at its intracanalicular genu or at the stylomastoid foramen, impairing function of these branches. Weakness of the ipsilateral depressor anguli oris can disrupt the muscular equilibrium of the floor of the mouth during tongue protrusion, thereby manifesting clinically as tongue deviation. The exact mechanism of this unusual facial nerve involvement remains speculative but could involve such stretch or compression.

Owing to the parents' insistence on retaining the external fixator, high-resolution magnetic resonance imaging could not be performed, thereby limiting direct visualization of cranial-nerve-vascular relationships and mechanistic dissection. Nonetheless, electroneurography unequivocally documented axonal impairment of the marginal mandibular and zygomatic divisions of the facial nerve, providing objective electrophysiological evidence implicating traction-mediated injury of these branches in the genesis of tongue deviation.

Upon recognition of neurological deterioration, traction load was immediately reduced, followed by a short pulse of corticosteroids and sustained neurotrophic therapy (mecobalamin and B-complex vitamins), supplemented by acupuncture. Gradual clinical recovery validates early decompression, targeted pharmacological support, and multimodal rehabilitation as an effective management algorithm for this rare complication.

Consequently, clinicians employing HPT must maintain heightened vigilance for potential cranial neuropathies—particularly those affecting the abducens (VI) and facial (VII) nerves. Clinical recommendations include: (1) serial neurological exams every 1–2 weeks during traction; (2) gradual traction increase; (3) immediate traction cessation upon symptom onset. These measures may prevent permanent injury.Systematic serial assessments of extra-ocular motility, facial symmetry, and tongue protrusion are imperative, complemented by prompt electrodiagnostic evaluation when aberrant findings are detected. Comprehensive pre-operative counselling should explicitly delineate these risks to optimize informed consent and recalibrate parental expectations. In summary, proactive recognition, stringent neuromonitoring, and immediate multimodal intervention are indispensable to safeguard both neurological integrity and overall therapeutic efficacy in patients undergoing HPT.

## Data Availability

The original contributions presented in the study are included in the article/Supplementary Material, further inquiries can be directed to the corresponding author.
